# GWAS and Machine Learning Screening of Genomic Determinants Underlying Host Adaptation in Swine and Chicken *Salmonella* Typhimurium Isolates

**DOI:** 10.3390/microorganisms14020293

**Published:** 2026-01-27

**Authors:** Yifan Liu, Yuhao Wang, Yaxi Wang, Xiao Liu, Shuang Wang, Yao Peng, Ziyu Liu, Zhenpeng Li, Xin Lu, Biao Kan

**Affiliations:** 1 School of Public Health, Shandong University, Jinan 250012, China; 15376183975@163.com (Y.L.);; 2 National Key Laboratory of Intelligent Tracking and Forecasting for Infectious Diseases, National Institute for Communicable Disease Control and Prevention, Chinese Center for Disease Control and Prevention, Beijing 102206, China; 3 Shunyi District Center for Disease Control and Prevention, Beijing 101300, China

**Keywords:** *Salmonella* Typhimurium, GWAS, random forest, virulence factors, antimicrobial resistance genes

## Abstract

*Salmonella* Typhimurium is a major zoonotic pathogen, with pigs and chickens serving as key reservoirs for human infection, yet the genomic determinants of its host adaptation remain incompletely understood. This study integrated comparative genomics, genome-wide association studies (GWASs), and interpretable machine learning on 1654 high-quality genomes of swine- and chicken-origin *S.* Typhimurium isolates to identify host-associated genetic features. Phylogenetic analysis revealed host-preferred lineages and significantly lower genetic diversity within chicken-adapted subpopulations. Meta-analysis identified distinct host-associated profiles of antimicrobial resistance genes (e.g., higher prevalence of *floR* and *bla_TEM-1_* in swine) and virulence factors (e.g., enrichment of *allB* and the yersiniabactin system in chickens). GWASs pinpointed 1878 host-associated genes and multiple SNPs/indels, functionally enriched in metabolism, regulation, and cell processes. A two-stage Random Forest model, built using the most contributory features, accurately discriminated between swine and chicken origins (AUC = 0.974). These findings systematically revealed the genomic signatures of host adaptation in *S.* Typhimurium, providing a prioritized set of candidate markers for experimental validation.

## 1. Introduction

*Salmonella* is one of the most common foodborne zoonotic pathogens. It is estimated that non-typhoidal *Salmonella* causes over 93 million cases of gastroenteritis globally each year, resulting in approximately 155,000 deaths [1]. In the European Union, *Salmonella* remains the second most commonly reported foodborne gastrointestinal infection in humans, with 79,703 confirmed cases of human salmonellosis in 2024, corresponding to a notification rate of 18.6 cases per 100,000 population [2]. Meanwhile, the assessment of *Salmonella* infections in the United States indicates that there are approximately 1.35 million cases of *Salmonella* infection, 26,500 hospitalizations, and 420 deaths each year, resulting in a total economic loss of several billion dollars [3]. Among more than 2,600 *Salmonella* serotypes, *S.* Typhimurium and *S.* Enteritidis are the two most frequently isolated serotypes in human infections, collectively responsible for about 40% of human salmonellosis cases [4].

*S.* Typhimurium is a broad-host-range serovar, capable of infecting a wide array of hosts such as humans, cattle, pigs, poultry, and wild birds [5,6]. Variants with distinct host preferences and pathogenicities have been identified within this serotype. Notable examples include pigeon-adapted variants (e.g., phage types DT2 and DT99) [7] and swine-associated lineages (e.g., U288 and ST34) [8], each exhibiting distinct genetic and phenotypic signatures of adaptation. This diversification is driven by adaptive evolution, involving genomic changes such as acquisition or loss of mobile genetic elements (MGEs), pseudogenization, single-nucleotide polymorphisms (SNPs), and indels, which collectively remodel metabolic pathways, virulence, and stress responses [5,9]. Beyond these core genomic changes, acquired traits such as antimicrobial resistance also contribute to host adaptation, with resistance profiles known to vary significantly by host species and geographic origin [10]. Predictive models, including Random Forest, have successfully identified accessory genes and resistance determinants as key features for distinguishing host-associated lineages [11].

Deciphering the genetic basis of host adaptation is essential for outbreak source attribution, zoonotic risk assessment, and targeted intervention. Whole-genome sequencing (WGS) has become a cornerstone for high-resolution clonal analysis in outbreak detection and trace-back investigations [5]. The advent of WGS, combined with machine learning (ML), has further advanced the field by enabling predictive modeling that integrates complex genomic features beyond phylogenetic clustering. For instance, Random Forest models have been applied to predict host sources or virulence phenotypes in *S.* Typhimurium using features such as functional variants [12], accessory genes and SNPs [11], and intergenic regions [13]. Such studies have also revealed evolutionary trade-offs, such as between plasmid-mediated antibiotic resistance and virulence [14]. Despite these advances, a gap remains in deeply dissecting the specific genetic determinants underlying adaptation to economically important reservoir hosts like pigs and chickens. Most predictive models are trained on multi-host datasets and may not capture the fine-grained, biologically relevant mutations and gene content differences that specifically differentiate these two major agricultural reservoirs. Furthermore, there remains a critical need for the systematic integration of information from other methods, including genome-wide association studies (GWAS), to filter and validate host-specific markers.

Pigs and chickens are among the most significant reservoirs for human salmonellosis [15,16]. Previous studies have observed that *S.* Typhimurium isolates from swine and chicken tend to form distinct clonal groups in core genome phylogenetic trees, suggesting potential host-specific adaptations and underlying genetic differences. This study utilizes high-quality genomes of *S.* Typhimurium isolates isolated from pigs and chickens. By integrating comparative genomics and interpretable machine learning approaches, we aim to identify host-associated genomic features (e.g., SNPs, Indels, virulence and resistance genes) and evaluate their contributions to host prediction. The goal is to select a set of high-weight, interpretable candidate genetic markers that can distinguish between swine and chicken origins. These findings will provide crucial genomic clues and experimental targets for further investigation into the molecular mechanisms of host adaptation in swine- and poultry-associated variants of *S.* Typhimurium.

## 2. Materials and Methods

### 2.1. Data Acquisition and Isolate Selection

All publicly available *Salmonella* *enterica* genome sequences (as of August 2024) were downloaded from the National Center for Biotechnology Information database. A rigorous screening process was applied to obtain high-quality genomic data, with the following inclusion criteria: (1) The ‘host’ field in the metadata isolates explicitly indicated chicken- or swine-related terms. (2) The genome assembly quality met the required standards. To ensure the reliability of subsequent analyses, stringent quality control was performed on the downloaded draft genomes: CheckM2 (v1.0.1) [17] was used to assess genome completeness and contamination, retaining isolates with completeness ≥ 95% and contamination ≤ 5%; FastANI (v1.33) [18] was used with *Salmonella* Typhimurium LT2 (GCA_000006945.2) as the reference genome for Average Nucleotide Identity (ANI) analysis, retaining isolates with ANI ≥ 95%; QUAST (v5.0.2) [19] was used to evaluate assembly quality, retaining genomes with N50 > 10,000 bp. (3) The in silico serotyping tool SISTR (v1.1.1) [20] was used to predict the serotype of all quality-controlled genomes, and only isolates predicted as *S*. Typhimurium were retained for subsequent analysis. Ultimately, this quality control pipeline yielded 1654 high-quality genomes, comprising 577 chicken-origin and 1077 swine-origin isolates. These isolates were initially collected from Asia, Europe, North America, Africa, South America, and Oceania. For the purpose of robust data analysis, regions contributing fewer than 100 isolates were consolidated into a single category, resulting in four final analytical groups: North America (1091 isolates; 65.96%), Europe (305; 18.44%), Asia (167; 10.10%), and Other (91; 5.50%).

### 2.2. Phylogenetic Analysis

To elucidate the phylogenetic relationships between chicken- and swine-origin isolates, the core genome of all isolates was aligned against the *S.* Typhimurium LT2 reference genome using the Snippy pipeline (v4.6.0) (https://github.com/tseemann/snippy, accessed on 30 July 2025) with its default parameters. The resulting core genome alignment file was processed with Gubbins (v3.4) (https://github.com/sanger-pathogens/Gubbins, accessed on 3 Augest 2025) using default settings to identify and filter recombination events. A Maximum Likelihood (ML) phylogenetic tree was constructed based on core genome SNP sites using IQ-TREE (v3.0.1) [21] software under the GTR nucleotide substitution model. Branch support was assessed with 1000 bootstrap replicates. The generated phylogenetic tree was visualized and annotated using the iTOL online tool (https://itol.embl.de/, accessed on 5 Augest 2025). To further resolve the fine-scale population structure, multi-level Bayesian clustering was performed on the core genome SNP alignment using RhierBAPS (v1.1.2) implemented in the rhierbaps R package, with default parameters.

### 2.3. Antimicrobial Resistance Gene (ARGs) and Virulence Factor (VFs) Analysis

The Resistance Gene Identifier (RGI) (v6.0.1) software was used with the CARD [22] database to predict ARGs. BLASTP (v2.13.0) [23] was used to compare sequences against VFDB [24] database, with an e-value threshold of 1e-5 and a per-hit query coverage threshold of 80% (-qcov_hsp_perc 80). To ensure reliability, a uniform standard was applied for identifying both ARGs and VFs: sequence identity ≥ 90% and coverage ≥ 80%.

Due to sampling size imbalances across geographical regions, a meta-analytic approach combined with permutation testing was implemented in R (v4.1.0) to enhance the reliability of estimating prevalence rates of ARGs and VFs in chicken and swine-origin strains. This was performed using a random-effects meta-analysis model using the meta package (version 8.2-1) in R to estimate the global prevalence rates for ARGs and virulence factors in *S.* Typhimurium isolates from two hosts (chicken and swine) across different continents. For each target gene, the prevalence rates were compared between chicken and swine origins, and a permutation test was applied to calculate *p*-values for the observed differences.

The specific procedure was as follows: (1) Random-effects Meta-analysis: for each target gene, the random-effects model was applied using the metaprop function to calculate the pooled prevalence for each host (chicken and swine) across continents, considering between-study heterogeneity. (2) Permutation Testing: for each target gene, a permutation test was employed to assess the significance of the difference in prevalence rate between chicken and swine. The observed difference in pooled prevalence rates was compared to a distribution of random differences obtained by shuffling the data (1000 iterations), with *p*-value calculated as the proportion of random differences greater than the observed difference. (3) Adjustment for Multiple Comparisons: the *p*-values were adjusted for multiple testing using the Benjamini-Hochberg (BH) method to control the false discovery rate.

### 2.4. Analysis of MGEs

We identified MGEs, including integrons, plasmids, and transposons, in all *S.* Typhimurium isolates. Integron and transposon structures were predicted using BacAnt (v3.4.0) [25] with default parameters. Plasmids were identified using geNomad (v1.11.0) [26] with the recommended databases and score thresholds.

### 2.5. GWAS

All 1654 genomes were annotated using Prokka (v1.14.5) [27] to generate standardized GFF3 files for downstream analysis. The pan-genome was constructed from the Prokka annotation results using Panta (v2.3.1) [28], which clusters homologous genes at a protein sequence identity threshold of 95%. The gene presence/absence matrix generated by Panta, along with host source information (chicken/swine) as the trait file, was input into Scoary (v1.6.16) [29] for association analysis. Genes with a Bonferroni-corrected *p*-value < 0.05 were retained as candidate genes significantly associated with the host.

SNPs and Indels in the isolate genomes were called using Snippy. To perform association analysis, these variants were first converted into presence/absence matrices. These matrices, alongside host source information, were then used as input for Scoary to identify variant loci significantly associated with host origin. Loci with a Bonferroni-adjusted *p*-value < 0.05 were considered significant.

For each set of features (genes, SNPs, and Indels) that passed an initial significance threshold (Bonferroni-corrected *p*-value < 0.05) in Scoary, we applied the same meta-analytic framework combined with a permutation test described in Section 2.3. Briefly, for each candidate feature, a random-effects meta-analysis was performed to estimate its pooled prevalence in chicken- and swine-origin isolates across continents, followed by a permutation test (1000 iterations) to assess the significance of the observed prevalence difference. The resulting *p*-values were adjusted using the Benjamini-Hochberg method. Only features that remained significant (adjusted *p*-value < 0.05) were retained as the final set of high-confidence, host-associated genetic markers.

### 2.6. Functional Annotation and Enrichment Analysis

Protein sequences corresponding to significant genes identified by GWAS, and SNPs/Indels located within coding sequences, were annotated for KO and COG functional classifications using eggNOG-mapper (v2.1.12) [30]. Based on the annotation results, the distribution of COG functional categories was visualized using the ggplot2 package in R.

### 2.7. Random Forest Model Construction and Evaluation

#### 2.7.1. Model Framework and Overall Strategy

Model construction was divided into two stages. The first stage aimed to distinguish isolates originating from chickens or swine from all isolates of other origins. The second stage specifically distinguished between chicken and swine origins, given that the isolate was predicted as being from one of these two hosts. Using the tidymodels framework in R, a stratified nested cross-validation strategy was employed for model building and evaluation to prevent data leakage and overfitting. The outer loop performed 5-fold cross-validation, while the inner loop performed 3-fold cross-validation on the training set for feature selection and hyperparameter tuning.

#### 2.7.2. Feature Selection and Hyperparameter Tuning

Feature selection was performed within the nested cross-validation, and hyperparameter selection was conducted in the inner cross-validation to ensure robustness and prevent data leakage. For the first-stage model, the initial feature pool was the gene presence/absence matrix from the pan-genome analysis. A three-step feature selection pipeline was applied: (1) GWAS-based screening using Scoary, which was run independently on each training set of the outer cross-validation folds; (2) refinement via Elastic Net regression using the glmnet R package (v4.1-3); (3) final selection using Recursive Feature Elimination with a Random Forest classifier. The Random Forest model was built using the ranger package (v0.14.1) within the tidymodels framework (v1.1.0) in R. Hyperparameters (mtry, trees, and min_n) were optimized using a grid search method. Model evaluation was performed using the test sets from the outer loop of the nested cross-validation. The second-stage model integrated multiple types of genomic features, including genes, SNPs, and Indels. The same methods as the first-stage model were used for model construction and evaluation.

#### 2.7.3. Model Evaluation

The final model performance was evaluated using the test sets from the outer cross-validation loop. Evaluation metrics included the Area Under the Receiver Operating Characteristic Curve (AUC-ROC), Balanced Accuracy, Precision, Recall, and F1-score.

#### 2.7.4. Feature Importance Analysis

To interpret the model and identify feature contributions, SHAP values were calculated using the fastshap package in R. The shapviz package was used to generate beeswarm plots and feature importance bar charts, visually illustrating the impact direction and relative importance of each feature on the model predictions.

## 3. Results

### 3.1. Population Structure and Phylogeny

**Figure 1:**
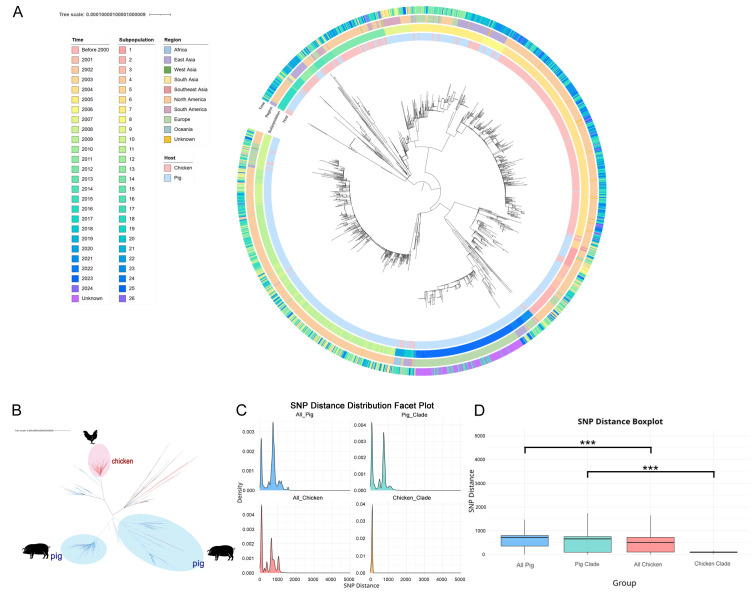
(**A**) Population structure of *S.* Typhimurium and its association with host species. Maximum likelihood phylogeny of *S.* Typhimurium strains was constructed based on core genome sequence variations. The phylogenetic tree is rooted at the center. The first to fourth concentric rings from the inside represent the host source, subpopulation, geographic region, and isolation year of the *S.* Typhimurium strains, respectively. (**B**) Unrooted phylogenetic tree of *S*. Typhimurium constructed based on core genome single-nucleotide polymorphisms (SNPs). Monophyletic clades predominantly consisting of chicken-origin isolates are highlighted in red, and those predominantly consisting of swine-origin isolates are highlighted in blue. (**C**) Density distributions of SNP distances among *Salmonella* isolates from different host sources. This panel shows the probability density distributions of pairwise SNP distances within four groups: all swine-origin isolates (blue), swine-origin monophyletic clade isolates (cyan), all chicken-origin isolates (red), and chicken-origin monophyletic clade isolates (orange). The X-axis shows SNP distance, and the Y-axis shows density. To clearly display the primary data distribution, the X-axis range covers the main distributional range (1st to 99th percentiles) of the data. Clustering of density curves at lower SNP distances indicates higher genetic homogeneity within a group, whereas broader distributions indicate greater genetic diversity. (**D**) Boxplot comparison of SNP distances between groups of *S.* Typhimurium isolates. This boxplot summarizes the distribution of pairwise SNP distances within the four groups: All Swine (blue), Swine Monophyletic Clade (cyan), All Chicken (red), and Chicken Monophyletic Clade (orange). Each box shows the median (center line), interquartile range (box boundaries), and the data range. There are significant differences among the groups, and these differences are indicated by asterisks (***, *p* < 0.001).

The isolates included in this study exhibited wide diversity in terms of temporal and geographical distribution. The isolation years of the isolates spanned over five decades, from 1968 to 2024. The isolates originated from all continents except Antarctica, with the majority sourced from North America (primarily the United States) and East Asia (primarily China) (Figure 1A).

The Maximum Likelihood phylogenetic tree constructed based on core genome-wide SNPs revealed that the chicken- and swine-origin *S.* Typhimurium isolates in this study formed at least five major lineages containing a relatively large number of isolates, although some clades exhibited substantial genomic divergence. Among these, three lineages were predominantly composed of either chicken- or swine-origin isolates, forming distinct clusters with a strong preference for either chicken or swine (Figure 1B). To further resolve the fine-scale population structure, we performed multi-level Bayesian clustering analysis using RhierBAPS, which identified 26 subpopulations. Several of these subpopulations demonstrated strong host specificity. For instance, Subpopulation 6 (n = 358) consisted of 99.2% chicken-origin isolates, forming a distinct “chicken-adapted lineage”. In contrast, Subpopulations 3, 4, 5, 9, 12, 24, and 25 (n = 291) were exclusively composed of swine-origin isolates (100%), collectively forming “swine-adapted lineage”. The swine-adapted lineage consisting of multiple subpopulations indicates multiple independent host adaptation events within the lineage, leading to the formation of genetically discrete subpopulations with specialized host preferences (Table S1).

To quantify the differences in population genetic diversity between chicken- and swine-origin isolates, we first compared the core genome SNP distances. The overall distributions of SNP distances were significantly different between all chicken-origin and all swine-origin isolates (Median: 577 vs. 720, D = 0.294, *p* < 2.2 × 10^−16^, Kolmogorov-Smirnov test). A significant difference was also observed between the chicken-adapted and swine-adapted subpopulations (Median: 95 vs. 675, D = 0.656, *p* < 2.2 × 10^−16^) (Figure 1C,D). This statistically confirms that the genetic diversity of the chicken-origin population, particularly the chicken-adapted subpopulation, is significantly lower than that of the swine-origin population.

### 3.2. Analysis of Differences in ARGs

**Figure 2:**
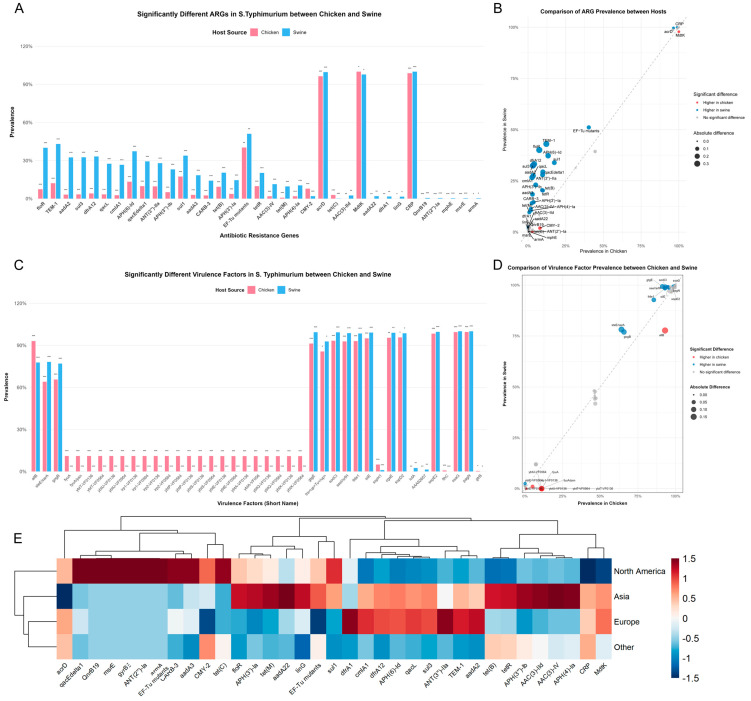
Comparative analysis ofARGs and VFs between swine- and chicken-origin *S*. Typhimurium isolates. (**A**) Significantly different ARGs. Grouped bar chart displays the prevalence of ARGs identified as having a statistically significant differential distribution between swine- and chicken-origin isolates (adjusted *p*-value < 0.05). Genes are sorted by the magnitude of the prevalence difference. The y-axis shows the global pooled prevalence (%), and bars are colored by host source (blue: swine; red: chicken). Asterisks above the bars indicate the significance level of the difference (***, adjusted *p* < 0.001; **, adjusted *p* < 0.01; *, adjusted *p* < 0.05). (**B**) Scatter plot of ARG prevalence. Dot plot compares the global pooled prevalence of all analyzed ARGs between chicken (x-axis) and swine (y-axis) hosts. The dashed diagonal line indicates equality. Points are colored based on significant host enrichment (blue: significantly higher in swine; red: significantly higher in chicken; gray: no significant difference). Point size corresponds to the absolute difference in prevalence. A subset of significantly different ARGs is labeled. (**C**) Significantly different VFs. Grouped bar chart displays the prevalence of VFs identified as having a statistically significant differential distribution between hosts (adjusted *p*-value < 0.05). VFs are represented by their abbreviated names (extracted from parentheses in the full annotation) and sorted by the magnitude of the prevalence difference. Coloring and significance notation follow the same scheme as panel A. (**D**) Scatter plot of VF prevalence. Dot plot compares the global pooled prevalence of all analyzed VFs between chicken and swine hosts. Plotting conventions (axes, diagonal line, point color, size, and labeling) are identical to those described for panel B. (**E**) Geographic distribution of host-associated ARGs. Heatmap illustrates the normalized carriage rates of the significant ARGs (from panel A) across major geographic regions. Rows represent regions, and columns represent ARGs. The color gradient (red-blue) indicates values above (red) or below (blue) the average carriage rate for each gene after row-wise Z-score normalization. Dendrograms show hierarchical clustering of regions (row) and ARGs (column) based on Euclidean distance and complete linkage.

A comparison of the number of ARGs carried by isolates from the two sources revealed that swine-origin isolates harbored a significantly higher number of ARGs than chicken-origin isolates (*p* < 0.001). We further identified 35 ARGs (Table S2) with significantly different evaluated prevalence rates between the two hosts using a meta-analytic approach combined with a permutation test (adjusted *p* < 0.05) (Figure 2A,B). The most pronounced disparities were observed for genes conferring resistance to aminoglycosides, sulfonamides, phenicols, and beta-lactams. The *floR* gene (chloramphenicol resistance) exhibited a markedly higher prevalence in swine-origin isolates (40.1%) compared to chicken isolates (7.4%), representing a prevalence difference of 32.6% (adjusted *p* < 0.001). Similarly, the beta-lactamase gene *bla_TEM-1_* was significantly more prevalent in swine (43.0%) than in chickens (12.1%, Δ = 30.9%, adjusted *p* < 0.001). This pattern extended to several other high-prevalence ARGs in swine, including *aadA2* (aminoglycoside, Δ = 29.3%), *sul3* (sulfonamide, Δ = 29.2%), and *dfrA12* (trimethoprim, Δ = 29.1%), all with adjusted *p* values < 0.001. Conversely, a smaller subset of genes showed a higher prevalence in chicken-origin isolates. The beta-lactamase gene *bla_CMY-2_* was detected in 7.9% of chicken-origin isolates versus only 2.0% of swine-origin isolates (Δ = −5.9%, adjusted *p* < 0.001). The tetracycline resistance gene *tet(C)* was also more common in chickens (2.9% vs. 0.2% in swine, Δ = −2.7%, adjusted *p* < 0.001).

To investigate the potential transmission mechanisms underlying these host-differentiated ARGs, we analyzed their associations with MGEs and co-occurrence patterns. Of the 1077 swine and 577 chicken isolates in our dataset, 719 (66.8%) and 139 (24.1%), respectively, carried at least one of the 35 host-differentiated ARGs located on a detectable MGE and were thus included in the co-occurrence analysis. Swine-origin isolates exhibited significantly more ARG-MGE associations per isolate than chicken-origin isolates (median: 4 vs. 2; Wilcoxon rank-sum test: *p* < 0.001). Among these, combinations in swine isolates involved more genes on average. The most prevalent resistance combination, *APH(3″)-Ib;APH(6)-Id* on transposons, was found in 23.6% of swine isolates with MGE-associated ARGs (n = 170) and 16.6% of corresponding chicken isolates (n = 23). Other high-frequency, multi-gene combinations were almost exclusively observed in swine, such as the integron-associated *aadA2;ANT(3″)-IIa;cmlA1;qacL* (12.4% of swine isolates, n = 89) and the transposon-associated *APH(3″)-Ib;APH(6)-Id;TEM-1* (11.7%, n = 84). A complete list of co-occurring ARG combinations is provided in Table S3.

Analysis of the geographical distribution of the significantly differential ARGs revealed a complex pattern associated with both host source and geographic region (Figure 2E). In North American isolates, genes such as *qacEdelta1* (quaternary ammonium compound resistance), *sul1* (sulfonamide), and *CARB-3* (beta-lactam) were notably enriched relative to other regions. Asian isolates were characterized by higher relative prevalence of *floR*, *APH(4)-Ia* (aminoglycoside), *AAC(3)-IV* (aminoglycoside), and *dfrA12*. In contrast, European isolates showed distinct enrichment for *bla_TEM-1_*, *ANT(3″)-IIa* (aminoglycoside), *cmlA1* (phenicol), and *aadA2*.

### 3.3. Analysis of Differences in Virulence Factor Profiles

Beyond antimicrobial resistance, we investigated the distribution of VFs between chicken- and swine-origin *S*. Typhimurium isolates using a rigorous meta-analytic approach. Permutation test with Benjamini-Hochberg correction identified 41 VFs (Table S4) with significantly different prevalence between the two hosts (adjusted *p* < 0.05) (Figure 2C,D). The virulence repertoire displayed distinct host-associated patterns, with the most pronounced differences observed in nutritional factors and accessory effector proteins.

Several virulence determinants exhibited substantially higher prevalence in chicken isolates. The most notable difference was observed for the allantoinase gene *allB*, involved in nitrogen utilization, which was detected in 93.1% of chicken isolates compared to 77.7% of swine isolates (Δ = 15.4%, adjusted *p* < 0.001). Anti-inflammatory type III secretion system (T3SS) effectors, SteE/SarA and GogB, were also significantly more common in chicken isolates, with prevalence differences of 14.1% and 11.2%, respectively (adjusted *p* < 0.001). Furthermore, the complete yersiniabactin siderophore biosynthesis, transport, and receptor machinery (e.g., *fyuA*, *irp1*, *irp2*, *ybtS*) was highly enriched in chicken isolates, with a pooled prevalence of approximately 11.0% across all related genes. In stark contrast, these genes were nearly absent in swine-origin isolates (prevalence ~0.01%). Further analysis of the phylogenetic distribution revealed that the yersiniabactin system was almost exclusively confined to the chicken-adapted Subpopulation 6, where it was detected in 58.3% (207/355) of isolates. In contrast, other chicken-enriched VFs, such as *allB*, exhibited a broader distribution across multiple chicken-associated subpopulations. Several other T3SS and type VI secretion system (T6SS) effectors, including GtgE, SodCI, SseI/SrfH, and Tre(Tu), also showed a higher, though more modest, prevalence in chicken isolates (Δranging from 5.9% to 8.1%, adjusted *p* < 0.001 for most).

Conversely, a distinct set of VFs was more prevalent in swine isolates. The aerobactin receptor gene *iutA* was exclusively identified in swine isolates (2.5% prevalence, adjusted *p* = 0.006). The curli production assembly protein CsgE and a putative invasion-associated protein (AAA92657) were also found at significantly higher frequencies in swine (Δ = 3.5% and 1.4%, respectively; adjusted *p* = 0.006 for both). Additionally, several core invasion and effector proteins, such as the adhesin/invasin PagN and the T3SS needle protein SsaG, were present at near-ubiquitous levels in both hosts but exhibited statistically significant, slightly higher prevalence in swine (100% vs. 99.5–99.9% in chickens, adjusted *p* < 0.001). Other VFs with adjusted significance included the LPS modification enzyme GtrB (exclusive to chickens, 0.4% prevalence, adjusted *p* = 0.029) and the SPI-2 effector SopD2 (more prevalent in chickens, Δ = 2.9%, adjusted *p* = 0.044).

### 3.4. Host-Specific Genetic Markers Revealed by GWAS

Association analysis of the gene presence/absence matrix with host source using Scoary identified a total of 2252 genes (Table S5) significantly associated with host (Bonferroni_*p* < 0.05). After a meta-analytic procedure combined with permutation test filtering, a final set of 1878 genes was identified. The vast majority of these genes were significantly enriched in chicken-origin isolates, and most encoded hypothetical proteins of unknown function. KEGG annotation analysis revealed that the most frequently occurring gene was associated with Ko:K08151 (12 occurrences), identified as tetracycline efflux pump (tetA) of Major Facilitator Superfamily (MFS). Other highly enriched genes included: Ko:K00984 (11 occurrences, aminoglycoside adenyltransferase aadA4, conferring resistance to streptomycin and spectinomycin), Ko:K18476 (11 occurrences, tetracycline repressor protein tetR), Ko:K01571 (10 occurrences, oxaloacetate decarboxylase oadA, involved in pyruvate metabolism and energy generation), and Ko:K03497 (10 occurrences, chromosome partitioning protein parB). Additionally, Ko:K07484 (10 occurrences) was identified as an IS66 family transposase, suggesting a potential role for MGEs in host adaptation.

**Figure 3:**
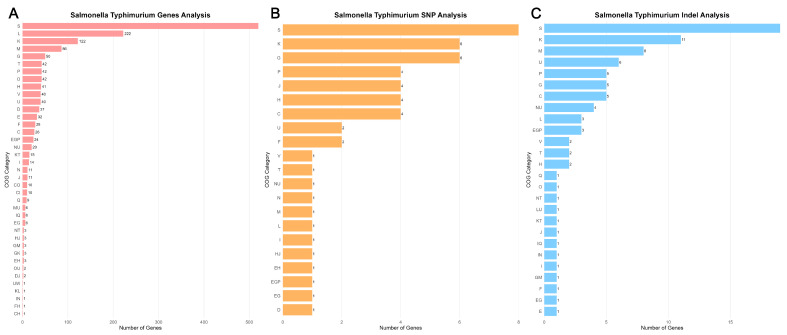
Functional distribution of *S*. Typhimurium genes based on Clusters of Orthologous Groups (COG) classification for gene, SNP, and Indel analyses. The horizontal bar chart displays the distribution of genes across different COG functional categories. The x-axis represents the number of genes assigned to each COG category, and the y-axis lists the functional categories along with their corresponding descriptions. Each bar is annotated with the exact gene count. COG categories are denoted by single-letter codes with their functional associations as follows: J, Translation; K, Transcription; L, Replication and repair; V, Defense mechanisms; M, Cell wall/membrane/envelope biogenesis; T, Signal transduction mechanisms; and other indicated categories. Multiple letter combinations represent genes assigned to multiple functional categories.

COG functional category statistics further revealed the global functional distribution of host-associated genes (Figure 3A). The 1878 host-associated genes were widely distributed across 20 functional categories. Category S (Function unknown) constituted the largest group (520 genes). Other significantly enriched categories included L (Replication, recombination and repair, 222 genes), K (Transcription, 122 genes), and M (Cell wall/membrane/envelope biogenesis, 84 genes). Notably, categories closely related to virulence and environmental adaptation were also significantly enriched, including V (Defense mechanisms, 40 genes), *p* (Inorganic ion transport and metabolism, 42 genes), and T (Signal transduction mechanisms, 42 genes), indicating that host adaptation involves sophisticated environmental sensing and stress response mechanisms.

In the SNP analysis, 174 loci (Table S6) were significantly associated with the host, of which 54 were located within coding sequence (CDS) regions. KEGG annotation showed that, aside from 12 loci that could not be definitively annotated, the associated genes were primarily involved in basic metabolic functions: Ko:K00426 (Cytochrome bd ubiquinol oxidase subunit II), Ko:K00768 (Cobalamin biosynthesis protein CobT), and Ko:K00845/K00885 (N-acetylmannosamine kinase NanK), each appearing once. COG functional classification statistics indicated that these SNP-associated genes were significantly enriched in categories S (Unknown function, 9 genes), K (Transcription, 6 genes), and G (Carbohydrate transport and metabolism, 6 genes) (Figure 3B), suggesting that host-specific SNPs may influence bacterial fitness by regulating gene expression and carbohydrate utilization.

In the Indel analysis, 290 significantly associated loci (Table S7) were identified, with 100 located in CDS regions. KEGG annotation results showed that Ko:K18326 (Transmembrane secreted effector protein HsrA) had the highest occurrence frequency (2 times). Others, such as Ko:K00005/K00096 (Iron-dependent alcohol dehydrogenase GldA) and Ko:K00104/K10530 (FMN-dependent dehydrogenase), each appeared only once. The COG functional distribution showed that Indel-associated genes were also predominantly in category S (Unknown function, 21 genes). Furthermore, categories K (Transcription, 11 genes), M (Cell wall/membrane/envelope biogenesis, 8 genes), and U (Intracellular trafficking, secretion, and vesicular transport, 6 genes) were significantly enriched (Figure 3C). It is particularly noteworthy that category V (Defense mechanisms) genes, associated with virulence and defense mechanisms, were also enriched, further supporting the notion that host adaptation is a complex process involving multi-layered interactions.

### 3.5. Random Forest Model for Predicting Chicken and Swine Host Sources of S. Typhimurium Based on Genome Characteristics

**Figure 4:**
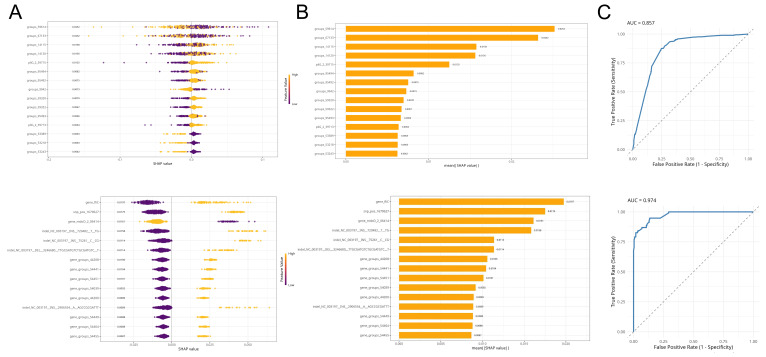
Evaluation and interpretability analysis of the Random Forest model. (**A**) Beeswarm plot of feature importance; (**B**) Bar plot of feature importance; (**C**) Receiver operating characteristic (ROC) curves of the models; SHAP, SHapley Additive exPlanations; AUC, area under the curve.

The objective of the first-stage model was to distinguish chicken- or swine-origin isolates from isolates of other potential sources. This stage ultimately selected a feature subset comprising 130 key genetic markers. Model evaluation based on the test sets from the outer 5-fold cross-validation demonstrated reliable discriminatory power, as shown by the ROC curve in Figure 4C (top), with an area under the curve (AUC) of 0.857. The model exhibited high sensitivity (0.893) in identifying chicken/swine-origin isolates, indicating its effectiveness in capturing isolates belonging to the target hosts. The model’s precision, F1-score, and balanced accuracy were 0.772, 0.823, and 0.755, respectively. Collectively, these metrics indicate that the first-stage model performs the initial screening task with acceptable efficacy, providing a reliable foundation for the fine-grained discrimination in the second stage.

The second-stage model performed fine-grained discrimination between chicken- and swine-origin isolates. After feature selection, the model ultimately retained 78 optimal key genetic markers. Analysis based on SHAP (SHapley Additive exPlanations) values revealed the contribution of each feature to the model’s performance (Figure 4B). Features with high contribution included SNP loci pos_1679627, Indel markers INS_720482_T_TG and INS_75261_C_CG, and the gene cluster *fliC* (Table 1). The beeswarm plot (Figure 4A) further illustrated the direction of feature influence on the prediction outcome: positive SHAP values indicate that the presence or higher expression of a feature makes the model more likely to predict the isolate as originating from a chicken host.

Evaluation metrics calculated from the confusion matrix based on the outer 5-fold cross-validation test sets yielded a sensitivity of 0.835, specificity of 0.966, precision of 0.929, F1-score of 0.880, and a balanced accuracy of 0.901. These metrics collectively demonstrate that the model distinguishes chicken-origin from swine-origin isolates with high accuracy and maintains high efficacy for predictions in both host classes, indicating an overall good fit. Furthermore, the ROC curve was plotted to visualize model performance across different classification thresholds (Figure 4C). An Area Under the ROC Curve (AUC) value of 0.974 was achieved, demonstrating the model’s high-reliability discriminatory power in accurately predicting the host source of *Salmonella* Typhimurium from their genomic data.

**Table 1:** Top 15 Features Ranked by SHAP Values in the Random Forest Model.

Features	Type	SHAP Score	Function
fliC	gene	0.0197	FliC/FljB family flagellin
pos_1679627	region	0.0175	/
mdoD_2_06414	gene	0.0161	glucan biosynthesis protein D
INS_720482_T_TG	CDS	0.0158	putative cytoplasmic protein
INS_75261_C_CG	CDS	0.0114	putative viral protein
DEL_3246680_TTGCGATGTCTGCGATGTC_T	region	0.0114	/
groups_44208	gene	0.0106	hypothetical protein
groups_54441	gene	0.0104	hypothetical protein
groups_54451	gene	0.0101	hypothetical protein
groups_54039	gene	0.0092	hypothetical protein
groups_44200	gene	0.0089	ParB/RepB/Spo0J family partition protein
INS_2906554_A_AGCCGCGATTT	CDS	0.0089	putative integrase core domain protein
groups_54449	gene	0.0088	hypothetical protein
groups_54464	gene	0.0088	hypothetical protein
groups_54455	gene	0.0087	hypothetical protein

## 4. Discussion

This study systematically revealed potential adaptive genetic differences of *Salmonella* Typhimurium in two major agricultural animal hosts, chickens and swine, by integrating large-scale genomics, GWAS, and machine learning from a functional and evolutionary perspective. Our findings confirm host-preferential differentiation within the population structure of *S*. Typhimurium and identify genetic features potentially associated with host adaptation across multiple functional layers, including antimicrobial resistance, virulence, metabolism, and regulation.

Phylogenetic analysis indicated the formation of multiple chicken- or swine-origin lineages, suggesting multiple independent host adaptation events may have occurred at the core genome level, leading to the formation of genetically discrete subpopulations with host preferences. Analysis of the core genome revealed that chicken-adapted subpopulations possess lower genetic diversity compared to swine-adapted subpopulations. Similar to the global dissemination of pathogenic lineages of Streptococcus suis via live animal trade, noted in that study [31], the formation of chicken-adapted subpopulations in our study might stem from a “selective sweep” experienced within modernized, highly homogeneous poultry production systems [32], leading to the dominance of a particular advantageous genotype.

This study revealed a significant divergence in the ARG profiles between *S*. Typhimurium isolates originating from chickens and swine, even after controlling for potential geographic confounding factors using a meta-analytic approach. Swine-origin isolates harbored a significantly higher burden of ARGs overall, a pattern largely driven by the markedly elevated prevalence of specific genes conferring resistance to aminoglycosides (e.g., *aadA2*, *APH(6)-Id*), sulfonamides (*sul3*), phenicols (*floR*), and beta-lactams (*bla_TEM-1_*).

The pronounced enrichment of *floR* and *bla_TEM-1_* in swine isolates is particularly noteworthy. The *floR* gene, encoding resistance to chloramphenicol and florfenicol, is a hallmark of certain successful *S*. Typhimurium lineages [33]. It is frequently located within *Salmonella* Genomic Island 1 (SGI1) in phage types such as DT104 and U302, which have historically been associated with swine and cattle [34]. The co-enrichment of genes like *aadA2*, *sul1*, and *dfrA12* observed in our swine isolates further echoes the classic resistance gene constellations found on SGI1 or similar integrative and conjugative elements (ICEs) prevalent in swine-associated clones [35,36]. Similarly, the high prevalence of *bla_TEM-1_* in swine isolates may be linked to its common association with the European monophasic *S*. Typhimurium clone (ST34/ST19), which often carries chromosomal or plasmid-borne *bla_TEM-1_* as part of its ASSuT resistance pattern [37]. The intensive use of beta-lactams and phenicols in swine production for therapy and prophylaxis, as documented in various production systems [38], provides a plausible selective pressure for the maintenance and spread of these specific resistance determinants.

Conversely, the higher prevalence of the *bla_CMY-2_* gene in chicken-origin isolates aligns with a distinct epidemiological pathway. *bla_CMY-2_*, encoding an AmpC β-lactamase, is frequently plasmid-borne in *Salmonella* from poultry [39]. The global emergence of CMY-2-producing *Salmonella* in poultry has been closely linked to the historical use of ceftiofur in this sector [40,41]. Our finding that *tet(C)* was also more common in chicken isolates adds another layer to this host-specific pattern, as different tetracycline resistance genes show variable associations with host species and MGEs [42].

The geographic variation in ARG enrichment underscores that host adaptation signals are superimposed on region-specific epidemiological landscapes. The enrichment of *qacEdelta1*, *sul1*, and *CARB-3* in North American isolates may reflect local circulating plasmid or integron types. The European pattern, with high *bla_TEM-1_*, *ANT(3″)-IIa*, and *cmlA1*, is consistent with reports of widely disseminated monophasic and multidrug-resistant *S*. Typhimurium clones across European swine production [43]. These geographic differences likely stem from variations in antimicrobial use practices, trade of live animals, and the historical presence and persistence of specific successful bacterial clones.

The host and geographic differences we observed are therefore primarily attributable to the accessory resistome—genes often carried on MGEs, such as plasmids, transposons, and integrons. The strong associations between specific ARG profiles and host species suggest that distinct selective pressures within swine and chicken production ecosystems shape the acquisition and retention of these MGEs. Furthermore, the potential for co-resistance and co-selection is significant, as many of the enriched genes are often physically linked on the same MGEs, meaning selection from one antimicrobial class can maintain resistance to others [44].

Our analysis revealed a distinct host-associated stratification in the virulence gene repertoire of *S*. Typhimurium, with the most pronounced differences observed in genes involved in nutritional adaptation and modulation of host inflammation. The significant enrichment of the *allB* gene, responsible for allantoin utilization, in chicken isolates aligns with the unique purine metabolism and physiological environment of avian hosts [45]. The presence of *allB* may thus confer a fitness advantage in the poultry gut by enabling the use of allantoin as a nitrogen source, potentially explaining the observed higher prevalence in chicken-origin strains and supporting the notion that its absence can slightly decrease virulence in chickens [45]. Furthermore, a suite of anti-inflammatory type III secretion system effectors, notably steE/sarA and gogB, were significantly more prevalent in chicken isolates. These effectors, often encoded on prophages like Gifsy-1, are key mediators of host immune suppression. SteE functions by reprogramming host kinase activity to drive an anti-inflammatory state conducive to bacterial persistence [46,47]. Similarly, GogB interferes with host inflammatory signaling [48]. The presence of the yersiniabactin siderophore system in chicken isolates highlights a critical divergence in nutritional immunity strategies. This high-affinity iron acquisition system is likely a key adaptation to the iron-restricted environment of the avian host [49]. Beyond iron scavenging, yersiniabactin may offer additional fitness benefits, such as mitigating metal toxicity [50].

In contrast, swine-origin isolates showed a preference for alternative virulence determinants. The significant association of the aerobactin receptor gene *iutA* with swine isolates points to the utilization of a different siderophore system, possibly reflecting distinct iron sources or host sequestration mechanisms in porcine hosts. The higher prevalence of curli-related gene *csgE* in swine may indicate an enhanced role for biofilm formation or adherence in the porcine infection cycle. The subtle but statistically significant higher prevalence of core invasion apparatus components like *pagN* and *ssaG* in swine isolates could suggest a marginal quantitative advantage in host cell invasion or intracellular niche establishment in this host species [51,52].

GWAS analysis provides a more comprehensive view of the genetic basis underlying host adaptation in swine- and chicken-origin *S*. Typhimurium. Identification of 1878 host-associated genes, a large proportion of which are hypothetical proteins of unknown function, indicates significant gaps remain in our understanding of the mechanisms underlying *S*. Typhimurium host adaptation; these genes represent candidate targets for subsequent functional studies. Concurrently, the enrichment of genes related to replication, recombination and repair (COG L), transcription (COG K), and cell wall/membrane/envelope biogenesis (COG M) suggests that host adaptation may involve multiple biological processes, including maintenance of genetic information stability, regulation of gene expression, and remodeling of cell surface structures. For instance, among the COG M hits, we identified the outer membrane efflux protein oprJ. This protein is part of multi-drug efflux systems that in Pseudomonas aeruginosa and other Gram-negative pathogens contribute to antibiotic resistance and biofilm formation [53], a key stress-adaptation and persistence trait. Similarly, within COG V, several genes were annotated as beta-lactamases (e.g., bla with EC 3.5.2.6), representing a classic enzymatic defense system.

Association analyses at the SNP and Indel levels revealed mutations related to carbohydrate transport and metabolism (COG G). For instance, an SNP annotated to N-acetylmannosamine kinase (NanK) might affect the bacterium’s ability to utilize host-derived sialic acid [54], analogous to previous research on Escherichia coli utilizing host mucosal cell surface glycoproteins as carbon sources [55]. Subtle variations in such metabolic pathways could influence the colonization efficiency of the bacterium within specific host intestinal niches. Furthermore, mutations associated with transcription and signal transduction might fine-tune host adaptive phenotypes by regulating the expression levels of key virulence or metabolic genes.

The interpretable machine learning framework employed in this study provided a robust, data-driven approach to distill complex genomic data into a minimal set of high-confidence markers for host source prediction. The two-stage Random Forest model successfully addressed the hierarchical nature of the problem, first filtering for isolates likely originating from the two major agricultural hosts before performing fine-grained discrimination between swine and chicken sources. The high AUC (0.974) and balanced accuracy (0.901) of the second-stage model demonstrate that the genomic feature set, encompassing genes, SNPs, and indels, contains sufficient information to accurately predict host origin. This finding aligns with and extends previous work where Random Forest models have successfully identified host-specific signatures in S. Typhimurium using various genomic features [11,12,13], revealing insights into evolutionary adaptations [14]. More importantly, the use of SHAP values for model interpretation moves beyond mere predictive performance to offer biological insights. The top-ranked features, such as the fliC gene and several SNPs/indels in hypothetical proteins, highlight specific genetic loci where variation is most informative for distinguishing between host-adapted populations. This aligns with and validates the signals independently identified through GWAS, confirming that machine learning can effectively prioritize candidate determinants from a high-dimensional feature space. This framework establishes a methodology for identifying stable, lineage-informed genomic signatures. The identified marker set provides a prioritized list of candidates for downstream functional validation, such as through mutagenesis and host colonization experiments, to directly test their role in swine- or chicken-specific adaptation.

This study has several limitations. First, the reliance on publicly available genomes may introduce ascertainment bias, as surveillance sequencing often focuses on outbreak investigations or specific clinically relevant clones. This could skew estimates of lineage composition and the prevalence of associated genetic features. Second, although we employed a meta-analytic framework to pool estimates across geographic regions, the uneven distribution of samples means that some signals could be influenced by region-specific practices. Third, both GWASs and machine learning identify correlations, and the biological roles of top features, especially hypothetical genes, require further experimental validation.

## 5. Conclusions

This study systematically analyzed the genomic differences between swine- and chicken-origin *S*. Typhimurium isolates using comparative genomics and interpretable machine learning approaches. The results demonstrate significant genomic population divergence between isolates from these two host sources, leading to the formation of multiple genetic lineages with distinct host preferences. This study identified several genetic markers significantly associated with host origin. The two-stage random forest models exhibited high performance in host source discrimination, validating the effectiveness of the selected genetic markers. These genetic characteristics provide novel genomic insights into the host adaptive evolution of *S*. Typhimurium, establishing prioritized targets for subsequent experimental validation.

## Data Availability

The original contributions presented in this study are included in the article/Supplementary Material. Further inquiries can be directed to the corresponding authors.

## Supplementary Materials

The following supporting information can be downloaded at https://www.mdpi.com/article/10.3390/microorganisms14020293/s1, Table S1: Overview of the 1654 *S.* Typhimurium isolates used for comparative genomics and host adaptation analysis; Table S2: List of antimicrobial resistance genes with significantly different prevalence in chicken versus swine hosts; Table S3: List and frequencies of co-occurring antimicrobial resistance gene combinations on mobile genetic elements in chicken- and swine-origin *S.* Typhimurium; Table S4: List of virulence factors with significantly different prevalence in chicken versus swine hosts; Table S5: List of genes identified by Scoary as significantly associated with host source; Table S6: List of snps identified by Scoary as significantly associated with host source; Table S7: List of indels identified by Scoary as significantly associated with host source.
